# qPCR assays with dual-labeled probes for genotyping honey bee variants associated with varroa resistance

**DOI:** 10.1186/s12917-021-02886-x

**Published:** 2021-04-30

**Authors:** David Claeys Boúúaert, Mario Van Poucke, Lina De Smet, Wim Verbeke, Dirk C. de Graaf, Luc Peelman

**Affiliations:** 1 Laboratory of Molecular Entomology and Bee Pathology, Ghent University, Krijgslaan 281, B-9000 Ghent, Belgium; 2 Animal Genetics Laboratory, Ghent University, Heidestraat 19, B-9820 Merelbeke, Belgium; 3 Department of Agricultural Economics, Ghent University, Coupure links 653, B-9000 Ghent, Belgium

**Keywords:** Honey bee, *Varroa destructor*, Varroa resistance, Suppressed mite reproduction, Resilience, High-throughput DNA test

## Abstract

**Background:**

The varroa mite is one of the main causes of honey bee mortality. An important mechanism by which honey bees increase their resistance against this mite is the expression of suppressed mite reproduction. This trait describes the physiological inability of mites to produce viable offspring and was found associated with eight genomic variants in previous research.

**Results:**

This paper presents the development and validation of high-throughput qPCR assays with dual-labeled probes for discriminating these eight single-nucleotide variants. Amplicon sequences used for assay validation revealed additional variants in the primer/probe binding sites in four out of the eight assays. As for two of these the additional variants interfered with the genotyping outcome supplementary primers and/or probes were developed. Inclusion of these primers and probes in the assay mixes allowed for the correct genotyping of all eight variants of interest within our bee population.

**Conclusion:**

These outcomes underline the importance of checking for interfering variants in designing qPCR assays. Ultimately, the availability of this assay allows genotyping for the suppressed mite reproduction trait and paves the way for marker assisted selection in breeding programs.

## Background

Since the first occurrence of the *Varroa destructor* mite in the Western honey bee *Apis mellifera*, honey bee health has become tightly interwoven with the presence and abundance of this ectoparasitic mite [1]. Originally the varroa mite occurred in South-East Asia where it has a balanced host-parasite relationship with the Asian honey bee *Apis cerana* [1]. Arriving in Europe around the 80’s [2] the varroa mite encountered a large pool of susceptible hosts lacking natural resistance [3, 4]. In addition to feeding on the fat body of bees [5], the mite provides a new transmission pathway for viruses causing rising problems with virus infections and ultimately colony mortality [6]. The initial response of the beekeeping community to control the varroa mite was heavily reliant on chemicals [1]. Although being effective, these chemicals also harm honey bees [7] and include disadvantages such as the deposition of residues in hive products [8] and the prevention of co-evolutionary processes to create a stable host-parasite relationship [9]. A long-term solution overcoming these disadvantages is to find and select on honey bee traits linked with varroa resistance or tolerance [10]. Over the last decades several research and breeding programs discovered multiple of these traits [2, 11, 12].

One of the key traits linked with varroa resistance is suppressed mite reproduction (SMR) which describes the non-reproduction of varroa mites in honey bee drone pupae cells [7, 13]. The precise mechanisms behind SMR are still not fully understood. Possible hypotheses are a suppression of the varroa reproduction cycle by lower levels of juvenile hormone [14], alterations in a gene from the ecdysone pathway [15] or diminished production of the brood pheromone [13]. Varroa reproduction may also be influenced by variations in the genotype of the mite or in the physiological status of the brood cell [16, 17]. In order to initiate breeding programs on SMR it is important to start by screening honey bee populations for the presence of the trait [18]. As an alternative for performing elaborative phenotypic assays, genotypic information can greatly increase the scale at which local populations can be screened. In addition, genotypic information can provide crucial insight in the mechanisms underlying varroa resistance [19].

Since the publication of the honey bee genome in 2006 [20] many studies identified quantitative trait loci or single nucleotide variants (SNV) associated with different varroa resistance traits [19]. For SMR, eight single-nucleotide variants were discovered by Broeckx and colleagues [13] using a novel whole exome sequencing design. Of the variants discovered six were risk associated variants and two were protective variants. The present research describes the design and validation of eight dual-labeled probe based qPCR assays for the high-throughput genotyping of the SMR trait.

## Results

**Fig. 1:**
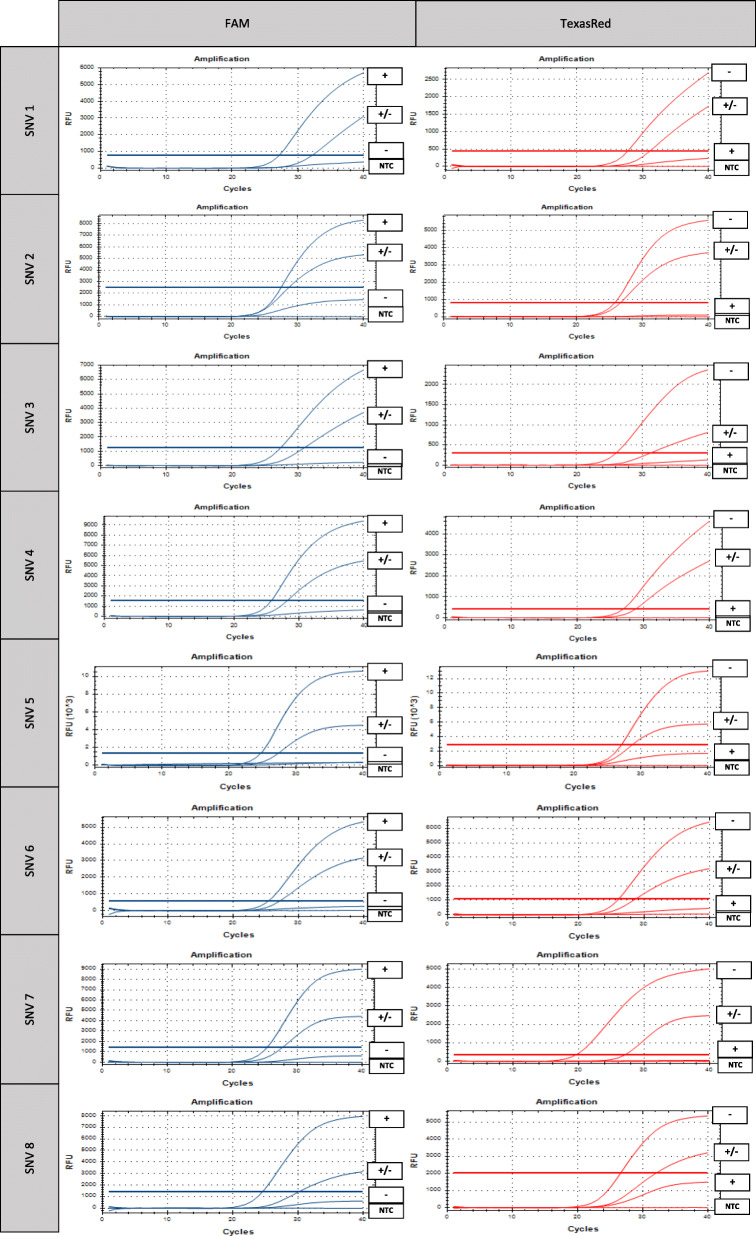
Amplification plots of the qPCR genotyping assays for the eight single-nucleotide variants (SNV) associated with suppressed mite reproduction. The left and right column show the plots for FAM and TexasRed signals, respectively. Homozygote wild type samples are indicated with +, heterozygote samples with +/−, homozygote variants type samples with – and the no template control with NTC. As shown in the plots, a correct distinguishment can be made between the absence or presence of both probe signals based on the threshold – determined based on the positive and negative controls – for both the homozygote and the heterozygote sample

Table 1 provides an overview of the primer and probe sequences of the qPCR assays used to genotype the eight SNVs associated with SMR, along with their amplicon lengths and optimal Ta. The resulting amplification plots are shown in Fig. 1. For the assays genotyping SNV 1, SNV 2, SNV 6 and SNV 8 no additional SNVs were present in the primer/probe binding sites in the 92 sequenced worker bees distributed throughout Flanders. For the assay genotyping SNV 3, a rare G > A variant was detected 3 bp downstream SNV 3 in the probe binding site of the Wt-allele in one bee, but it did not influence correct genotyping as the Wt-probe could still specifically bind to the Wt-allele and produce a strong signal, despite its single mismatch with the target. Similarly, for the assay genotyping SNV 7, a rare C > T variant was detected 3 bp upstream SNV 7 in the probe binding site of the Wt-allele in two bees, but did not influence correct genotyping either.

**Table 1:** Overview of the genotyped SNVs with the primer and probe sequences, amplicon lengths and annealing temperatures (Ta) of the qPCR assays. Target SNVs are indicated in bold, interfering SNVs are underlined

SNV	Nucleotide variant	Primer sequence	Probe sequence	Amplicon length	Ta
1	GB54921- RA:r.94G > A	F1: 5′-ACCCACTTTTTACTACGA-3′ R1: 5′-GCTTCTAGGCTGGATAA-3’	Wt1-probe: 5’-FAM-TGGACAAATTTA**C**CTTCTCGTTA-BHQ1–3′ Vt1-probe: 5′-TexasRed- TGGACAAATTTA**T**CTTCTCGT-BHQ2–3’	108 bp	58 °C
2	GB54921- RA:r.144 A > G	F1: 5‘-CCAAGTTCCCGTCAGA-3’ R1: 5′-TCGCCATTCTTCTCAGG-3’	Wt1-probe: 5’-FAM-CTCTAACGA**T**GCTTCTAGGC-BHQ1–3′ Vt1-probe: 5’-TexasRed-CTCTAACGA**C**GCTTCTAGGC-BHQ2–3’	106 bp	58 °C
3	GB47018- RA:r.1824C > U	F1: 5′-AAGGGACTAACTATAGCAAAA-3′ R1: 5′-GGCAGGAGGTGTTTTAG-3’	Wt1-probe: 5’-FAM-CGAATCGCT**C**CCGGAAA-BHQ1–3′ Vt1-probe: 5′-TexasRed-CGAATCGCT**T**CCGGAA-BHQ2–3’	90 bp	60 °C
4	GB53345- RA:r.37 A > GG	F1: 5’-AGCGATAAAATTTCTTCTTTCCTTA-3′ F2: 5′-AGCGATAAAATTTCTTCTTTCTTTATC-3′ R1: 5’-CATCGTCCTGGCGTAG-3’	Wt1-probe: 5’-FAM-AGCGTCA**T**CGCCGTC-BHQ1–3’ Vt1-probe: 5’-TexasRed-AGCGTCA**C**CGCCGTC-BHQ2–3’ Vt2-probe: 5’-TexasRed-CAGTGTCA**C**CGCCGTC-BHQ2–3’	118 bp	58 °C
5	GB53340- RA:r.4143 U > G	F1: 5′-CGAAGGTGGCCGAATTG-3′ R1: 5′-GCTTCTCCAACTCGTTCATC-3’	Wt1-probe: 5’-FAM-TCGGGAGGT**T**CTCATCCACC-BHQ1–3′ Vt1-probe: 5′-TexasRed-AATCGGGAGGT**G**CTCATCCA-BHQ2–3′ Vt2-probe: 5′-TexasRed-AATCGGGAGGT**G**CTGATCCA-BHQ2–3’	147 bp	60 °C
6	GB48382- RA:r.987 G > A	F1: 5‘-TGGCGAATGGGAAACAG-3’ R1: 5′-CTCGTACCTTTTCAGTCTTCA-3’	Wt1-probe: 5’-FAM-CGTTTATACG**C**GCCATTTTTCGA-BHQ1–3′ Vt1-probe: 5′-TexasRed-CGTTTATACG**T**GCCATTTTTCG-BHQ2–3’	132 bp	62 °C
7	GB50526- RA:r.1662G > A	F1: 5‘-CGTGATCGTCGGTGTTATC-3’ R1: 5′-GCGAGAGGGTGAAGGA-3’	Wt1-probe: 5’-FAM-TCTCCTTT**C**GGGTCGGCTG-BHQ1–3′ Vt1-probe: 5′-TexasRed-TCTCCTTT**T**GGGTCGGCT-BHQ2–3’	84 bp	62 °C
8	GB50114- RA:r.1662A > G	F1: 5‘-CTCTGAACACCCTGAACAAG-3’ R1: 5′-TCCAGCTCCTGTCCTTG-3’	Wt1-probe: 5’-FAM-TACTGCCCC**T**GGTGGC-BHQ1–3′ Vt1-probe: 5′-TexasRed-TTACTGCCCC**C**GGTGGC-BHQ2–3’	138 bp	62 °C

However, for the assay genotyping SNV 5, an abundant C > G variant was detected 3 bp downstream SNV 5 in the probe binding site of the Vt-allele in 57 bees, that prevented binding of the original Vt1-probe to the mismatch-containing Vt-allele and thus the detection of the Vt-allele. Adding the Vt2-probe to the assay, containing that additional variant, solved this problem. Most problematic was the assay genotyping SNV 4. A rare C > T variant (found in one bee) at the fourth last position of the F1-primer prevented amplification of the Vt-allele. In addition, an abundant G > A variant 5 bp downstream SNV 4 in the probe binding site of the Vt-allele (found in 35 bees) prevented the detection of the Vt-allele using the original Vt1-probe. Including the F2-primer and the Vt2-probe to the assay mix (both containing the corresponding additional variant), resulted in correct genotyping.

## Discussion

These results show that additional variants, which are common in honey bees due to their extremely high recombination rate [21], can lead to false results depending on their nature and position, as they can in all PCR-based techniques. Also, this finding underscores the importance of taking into account known variants from available sequence data or variant databases when designing assays and of checking results for anomalous amplification curves. Haploid samples (males) should always have a signal with only one probe. Diploid samples (females) should always have a typical homozygous signal (steep slope and high RFU-value) with one probe and no signal with the other probe, or a typical heterozygous signal (intermediate slope and intermediate RFU-value) with both probes [22]. Sequencing the complete qPCR amplicon region of samples with anomalous amplification plots with external primers, as described in Broeckx et al. [13], can identify unknown influencing variants.

Although sequencing is more informative, qPCR assays with dual-labeled probes allow for faster and cheaper high-throughput screening. For screening new populations, it is recommended to first sequence the qPCR amplicon regions in a small representative subset of that population and adjust the qPCR assays based on newly identified variants before using them for high-throughput screening. The assays can be performed on other qPCR platforms and reagents from other suppliers, but we advise to check the specific annealing temperature for every assay experimentally in the lab-specific setup with all positive controls and a no template control (NTC). In case of a lack of control samples, artificial oligos can be ordered containing the correctly orientated primer and probe sequences.

There are multiple options to tune up the assays. Assays can be run in the presence of fluorescent nucleic acid binding dyes such as SYBR green, to include melt curve analysis, useful to detect (non) specific amplification, oligo dimers, null-alleles or primer/probe-related problems during optimization, as described by Van Poucke et al. [23]. Assays performed at the same annealing temperature might be combined per two, if the probes of the second assay are differently labeled, e.g. with 5′-HEX-BHQ1–3′ and 5′-Cy5-BHQ2–3′. Although pooling techniques to detect allele ratios exist [24], it is difficult to precisely determine Wt/Vt allele ratios when working with more than two haploid drones or multiple diploid worker bees.

The use of genomic markers, such as the eight variants found by Broeckx et al. [13], in marker-assisted selection (MAS) is a promising method to accelerate the breeding progress on varroa resistance traits [25]. Thus far, only protein markers have been successfully applied in breeding programs in honey bees [26]. Compared to protein markers, genomic markers have the advantage of being independent of expression levels and are considered more stable [26]. The prerequisite however is that the high recombination rate in honey bees [21] does not cause the breakdown of inter-allele linkages through repeated rounds of meiosis [27]. Further research is ongoing to validate the effect of the eight SNVs on the SMR trait and thus of the applicability of genomic markers in MAS.

## Conclusion

Supporting ongoing selective breeding programs with honey bees by applying different ‘omics tools opens new possibilities for better understanding underlying mechanisms and unrolling marker-assisted selection programs [19]. The qPCR assays described in this paper neatly fits in with these future perspectives as it provides a novel laboratory based detection method to genotype honey bee colonies for the presence of the SMR trait.

## Methods

### Samples

Ninety-two DNA samples, used to analyze the allelic frequency of the eight SNVs in the Belgian honey bee population via Sanger sequencing in Broeckx et al. [13], were reused to optimize and validate the qPCR assays. They were isolated from two individual worker bees of the subspecies *A. m. carnica* from 46 different colonies located throughout Flanders, the northern part of Belgium.

### Assay design

A qPCR genotyping assay with dual-labeled probes was designed for each of the eight SNVs following the strategy described by Van Poucke et al. [22]. Depending on the amplicon sequence, probes were designed on the forward or the reverse strand. Wild type (Wt) probes were labeled with 5′-FAM and 3’-BHQ1, variant type (Vt) probes with 5′-TexasRed and 3′-BHQ2.

### Assay optimization and validation

The assays were performed in 10 μl containing 10x KEY buffer, 500 nM of each primer/probe, 800 μM dNTPs, 0.5 U TEMPase Hot Start DNA Polymerase (VWR) and 2 ng DNA on the CFX96 Touch Real-Time PCR Detection System (Bio-Rad). Thermal cycling conditions comprised 1 cycle of 14′40″ at 95 °C (activation Hot Start Polymerase and denaturation DNA) followed by 40 cycles of 20″ at 95 °C (denaturation DNA) and 40″ at the assay specific combined annealing/elongation/signal detection temperature (Table 1). Specific amplicon generation was checked by evaluating the PCR products using agarose gel electrophoresis. Optimal annealing temperature (Ta) was determined by performing gradient PCR and assessing probe specific signals on Wt/Wt (wild type homozygote), Wt/Vt (heterozygote) and Vt/Vt (variant type homozygote) samples. No template controls (NTC) were included to account for possible contaminations. The Sanger sequenced samples were used for validation and also checked for additional SNVs in the primer and probe binding sites. If present, they were evaluated for their influence on the result. For the assays genotyping SNV 4 and SNV 5 additional primers/probes had to be included for correct genotyping.

## Data Availability

All data generated or analyzed during this study are included in this published article.

## Abbreviations

SMR: Suppressed mite reproduction
SNV: Single nucleotide variant
